# Psychological Support in a COVID-19 Hospital: A Community Case Study

**DOI:** 10.3389/fpsyg.2021.820074

**Published:** 2022-02-17

**Authors:** Damiano Rizzi, Erika Asperges, Anna Rovati, Francesca Bigoni, Elena Pistillo, Angelo Corsico, Francesco Mojoli, Stefano Perlini, Raffaele Bruno

**Affiliations:** ^1^Fondazione Soleterre Strategie di Pace ONLUS, Milan, Italy; ^2^Unit of Emergency Medicine, Department of Internal Medicine, IRCCS Policlinico San Matteo Foundation, University of Pavia, Pavia, Italy; ^3^Unit of Infectious Disease, IRCCS Policlinico San Matteo Foundation, University of Pavia, Pavia, Italy; ^4^Unit of Respiratory Diseases, Department of Medical Sciences and Infective Diseases, IRCCS Policlinico San Matteo Foundation, University of Pavia, Milan, Italy; ^5^Unit of Anaesthesia and Intensive Care, Department of Clinical-Surgical, Diagnostic, and Pediatric Sciences, IRCCS Policlinico San Matteo Foundation, University of Pavia, Pavia, Italy

**Keywords:** psychological support, post-traumatic stress disorder, COVID-19, mental health, health care staff

## Abstract

Burnout is a well-documented entity in Care Workers population, affecting up to 50% of physicians, just as it is equally well established that managing an infectious disease outbreaks, such as confirmed in the COVID-19 pandemic, increases Post-Traumatic Stress Disorder (PTSD) and the psychological burden. Mental health support, in the form of formal or remote sessions, has been shown to be helpful to health care staff, despite the organizational difficulties in an emergency. During the first emergence of COVID-19 in Italy, the Scientific Institute for Research, Hospitalization and Health Care *Istituto di Ricovero e Cura a Carattere Scientifico (IRCCS) Policlinico San Matteo Foundation* (Pavia, Lombardy), the Italian hospital that treated “patient 1,” has activated an agreement with the *Soleterre Foundation*, an international Non-Governmental Organization (NGO) that manages health emergency projects, to provide psychological support. A task force of psychologists was created with the aim of designing and administering a *Therapeutic Mental Health Assessment for COVID-19 Care Workers* (TMHA COVID-19 CWs) to evaluate and support health care workers’ mental health. The assessment battery was developed to evaluate symptoms and behaviors associated with trauma and the corresponding maladaptive behaviors (the National Stressful Events Survey for PTSD-Short Scale “NSESSS” and the Diagnostic and Statistical Manual of Mental Disorders “DSM-5” Self-Rated Level 1 transversal Symptom Measure—Adult). Once the TMHA COVID-19 CWs had been developed, the team of psychologists regularly visited healthcare staff in the ward to administer it. One hundred seven care workers (44 males, mean age 40 ± 15) across Intensive Care Units (ICUs), the emergency room and medical ward were administered the TMHA COVID-19 CWs. PTSD symptoms were reported as severe by 13% of the population. Depressive symptoms as severe for 7% and Anxiety symptoms as severe for 14%. Severe psychotic symptoms were experienced by 2% and severe suicidal thoughts by 1% of the population. The possibility of acting upon the results of the TMHA COVID-19 CWs allowed an early intervention through individual session beyond the cut-off level (moderate and severe symptoms) for PTSD in NSESSS. In fact, 280 individual support sessions were offered. Therefore, we considered our project a protective and support factor for healthcare workers’ mental well-being and we recommend implementing a mental health screening program in ward involved in COVID-19 patients’ care.

## Introduction

The COVID-19 pandemic put medical personnel under unprecedented levels of stress. Burnout is already a well-documented entity in this population, affecting as many as 50% of physicians (West et al., 2018), and infectious disease outbreaks are known to increase the psychological burden because of overwork and fear of contagion and dealing with unknown diseases, new or unusual protocols and decreased resources (Walton et al., 2020). A review on risk factors for post-traumatic stress disorder (PTSD) symptoms after Coronavirus outbreaks, including COVID-19, demonstrated a high risk for PTSD among emergency health care staff, and an English study confirmed worrying rates of probable mental health disorders in ICU staff during the current pandemic (Carmassi et al., 2020; Greenberg et al., 2021).

Response to stress can be varied: from acute stress reaction to PTSD. Psychological support in the form of formal or remote (*via* telephone or computer) sessions have been shown to be helpful to staff, but they can be difficult to organize because of time constraints and contagion concerns (Maunder et al., 2003).

Italy was the second country, after China, to deal with a relevant number of COVID-19 cases, and since the very first days of the pandemic it became apparent that the evidence on worsening mental health of medical personnel during a pandemic was going to be confirmed. For this reason, in the first Italian hospital that received COVID-19 patients, the Scientific Institute for Research, Hospitalization and Health Care *Istituto di Ricovero e Cura a Carattere Scientifico (IRCCS) Policlinico San Matteo Foundation* (Pavia, Italy), the *Soleterre Foundation* decided to implement a program of psychological support for healthcare personnel.

Since 2015, *Soleterre Foundation* has an agreement protocol with the *IRCCS Policlinico San Matteo Foundation* and actively collaborates offering psychological support to pediatric patients, their families, doctors and health workers. *Soleterre* also has considerable experience as an international NGO in the management of health emergency in Europe and Africa^1^ including projects on psychological support and PTSD prevention in 22 middle and low income countries.^2^

As the health situation worsened, *Soleterre Foundation* offered to expand their collaboration within the hospital, making their psychologists available and adding external professionals, for both in presence and remote interventions.

In this regard, it should be noted that, although telepsychiatry has proven effective in various contexts (for example, psychiatric assessment, monitoring of interventions (Shore et al., 2007; Santesteban-Echarri et al., 2020), individual and group support for PTSD, anxiety or depression (Andersson et al., 2014; Berryhill et al., 2019a,b; Gentry et al., 2019; Liu et al., 2020), its effectiveness in emergency pandemic situations is still poorly understood. In fact, while the majority of hospitals in Italy activated their few clinical psychologists for remote sessions *via* telephone or video calls, *Soleterre Foundation* specifically aimed at working at least partially in the frontlines.

Thus, on the 16th March 2020, *Soleterre Foundation* and the hospital implemented an emergency psychological support project. The main aim was implementation of a task force of psychologists required to create and administer a tool to evaluate health care workers’ mental health. The objective of the intervention was to provide adequate psychological support where needed.

## Context

Since the outbreak of the COVID-19 emergency in Italy, in February 2020, *IRCCS Policlinico San Matteo Foundation*, a university hospital managing more than 1,000 beds, has been on the front line for COVID-19 patients from four different provinces. It has been declared by the Lombardy Region a COVID-19 hub hospital for the provinces of Pavia, Lodi, Cremona, and Milan’s southern metropolitan area, catering to a basin of 1.6 million inhabitants, with about 28,000 infections recorded in the first wave. During the first peak, 2,770 COVID-19 patients were admitted to the Emergency Room, 1,162 were admitted to the ward (including the Italian “patient 1”), and 397 patients died in-hospital over the course of 2 months. Actions had to be taken to accommodate the increased number of patients, many of them severely ill, including more than doubling the beds in the Infectious Diseases ward, creating an extra Emergency Room and ICU, converting or at least dedicating some beds of many other ward to COVID-19 (Asperges et al., 2020; Lenti et al., 2020; Perlini et al., 2020). The healthcare personnel was heavily involved in the emergency, and directly or indirectly exposed to the experience of death or the threat of death.

### Setting

Since the first days of the emergency, two Soleterre psychologists monitored the departments in order to understand the level of need in terms of psychological support and to estimate the number of psychologists to be activated.

From this work, an emergency project lasting 6 months (March–September 2020) was activated.

A task force of eleven clinical psychologists from the *Soleterre Foundation* active at *IRCCS Policlinico San Matteo Foundation* was set up to develop a mental health monitoring test for Care Workers *Therapeutic Mental Health Assessment for COVID-19 Care Workers* (TMHA COVID-19 CWs) and a psychological support intervention (in case of need) in continuity with local and national guidelines.

#### Targeted Sites

The target of the intervention were any healthcare personnel (physicians, nurses, nurse administrators, bed managers, and auxiliary nurses) working in the Emergency Room, the ICU and the ward of Infectious Diseases and Internal Medicine between the 16th of March and the 31st of May. Any staff that requested additional intervention was catered to. We excluded subjects who, while operating at *IRCCS Policlinico San Matteo Foundation*, were not in the job roles listed above or did not work in one of the departments listed above involved in the first line of the emergency at the time of filling in the questionnaires.

## Programmatic Elements

The first step was creating a task force: several meetings with the general management, the health management and the directors of the different departments involved within the *IRCCS Policlinico San Matteo Foundation* were necessary to recruit the members.

The TMHA COVID-19 CWs was then developed by borrowing the medical strategy for COVID-19 adopted and described by clinicians during clinical summits, starting from the following assumptions:

• A new disease needs a new approach, thus we need new clinical criteria; Like COVID-19’s natural history can be divided in two main phases (Siddiqi and Mehra, 2020) (a viral and a hyper inflammation phase), with some overlapping in the middle, we likewise represent two phases in mental symptomatology. The first is concurrent to the potential trauma (“viral” element) and characterized by specific symptoms. It is then followed by a potential readjustment (“immune response” element), but if no re-adaptation occurs, the onset of pathological symptoms linked to the specific life experience is very likely.
• The whole population, during a pandemic, is potentially affected by disorders related to life events and stressful conditions, including dissociative disorders (also following the historical relation between dissociation and conversion) maintained in the ICD-10.
• The intensity and quality of negative outcomes of a potentially traumatic situation are the result of the balance between the event’s characteristics and protection’s factors.

Typical protection factors include:

(1) **Hyperactivation**, a permanent state of alert that aims at keeping things under control while the whole world loses elements of daily certainty. In such state the psychophysical system increases the level of excitement, amplifies the emotional instability, exaggerates fear or aggressiveness and manifests symptoms of hyperarousal.
(2) **Turning off**, a state of demotivation, withdrawal, loss of energy.
(3) **Dissociation**, a kind of interruption between us and the threatening event. In this dissociative behavior the psychophysical system acts a sort of interruption of the generally integrated functions of consciousness, memory, identity or awareness of the body, the self or the environment. In such a hypoactivity (hypoarousal) the psychophysical system lowers the level of excitement and attenuates the alarm reaction, which could cause hypersomnia and insensitivity to stress.

While these defensive behaviors are natural in a moment of alarm, it is necessary that they do not become chronic (to get chronic in a noradrenergic dysfunction), beyond the threat because they could turn from defenses into pathogenic behaviors outside the emergency. There will be, therefore, a passage from the potentially traumatic phase to the adaption phase, that will occur at a different time point for each individual or group.

On the basis of these assumptions and the aforementioned knowledge in the field, tools appropriate to evaluate symptoms and behaviors associated with the protective factors described above and the corresponding maladaptive behaviors were chosen to be used in the TMHA COVID-19 CWs. This tools are the National Stressful Events Survey PTSD Short Scale (NSESSS) and the DSM-5 Self-Rated Level 1 transversal Symptom Measure—Adult, which are part of the Italian DMS-5 and are widely used in the Italian context. They were chosen also to allow comparison of data at European and international level. The scales are designed for administration during the initial interview and to monitor the progress of psychological interviews. The scales can be used in stand-alone mode, i.e., the total score does not refer to a normative distribution for the definition of cut-offs, which are defined *a priori*. In the DSM-5 further information that clarifies their use in the Italian context is available.

Once the TMHA COVID-19 CWs had been developed, the team of psychologists regularly visited healthcare staff in the ward to administer it and to determine who should be prioritized when offering support.

In each of the departments two psychologists were deployed at the same time 7 days a week, for a total of six psychologists always present. The team had its operational base in a space isolated from the other ward and used also for in presence sessions. Each ward had a room for individual and group support session. During the first emergency phase, each psychologist present in the ward directly organized individual sessions of 45 min and group sessions of 1 h (3–15 people involved) that were guided by the questions present in the TMHA COVID-19 CWs. At the same time, to reach a greater number of operators and to provide additional psychological support space, Soleterre activated a psychological support telephone line which received reports and calls. According to the request, the healthcare personnel were supported directly over the phone or *via* video call (individual 45-min session) or oriented toward face-to-face session. All the sessions were based on the TMHA COVID-19 CWs and therefore had a fixed pattern. However, given the emergency conditions, psychoeducation techniques were also used (relaxation, listening, and restitution of coping techniques) which cannot be systematized in duration (from 20 min to 1 h) because of their different nature. Support sessions took place mostly during debriefing sessions and shift changes, in order to avoid interrupting work on the ward.

Data were the verified and transcribed into an electronic database to analyze them in approximately 175 h. The number of individual psychological support sessions were 207 (25 in video call), while group session were 27 (for a total of 280).

### Process Evaluation

During the implementation, a methodological review based on the data collected was carried on with the support of the Department of Brain and Behavioral Sciences of University of Pavia. The aim was to evaluate *in interim* the efficacy of the evaluation and intervention model, investigating further psychological dimensions that could be affected by the emergency (i.e., Caregiving) or protection factors (i.e., social support).

While no changes to the implementation and intervention protocol was carried on at the time, this led to the addition of other tests and the development of a protocol for randomized controlled study on various modes of psychological intervention (remote vs. in person vs. no intervention) on healthcare staff for COVID19-related trauma (study in progress since February 2021).

Fidelity to the project was maintained throughout the study period.

### Economic Evaluation

The project in the emergency phase had a total cost of 78.311,00 €:

• € 19.000,00: Soleterre project management—coordination of actions and operations center,
• € 42.966,00: Psychological support—psychologists team for face-to-face and remote meetings,
• € 16.346,00: Personal protective equipment and materials for the project (printer, flyers, badges, stationery, telephony).

### Sample Size

Sample size was not considered in advance, as the service was provided during an emergency situation. Moreover, our objective was not to generalize our findings to the population but to provide support when requested; in fact the size and flexibility of the psychologists’ team allowed the service to be available whenever needed in accord with the health care staff’s schedule. However, a post-hoc analysis based on the prevalence of PTSD from the literature (Greenberg et al., 2021) reveals a power higher than 80%.

A target number of individual sessions to be administered when required was not pre-specified, as the aim was to provide support as needed.

### Analysis

Analysis was conducted retrospectively. Descriptive statistics was employed to summarize the findings.

### Ethical Approval

*IRCCS Policlinico San Matteo Foundation’s* ethical board committee gave its approval of the project (protocol number 20210057600). The approval was requested and obtained for the publication of the data after the implementation was carried out.

### Consent

All healthcare staff involved in the study provided their written consent or oral consent when the emergency situation prevented the acquisition of a written one, since the reorganization of the healthcare facilities had substantial repercussions on the standard procedures for collecting informed consent. During the administration of the tests we tried to respect standards of administration in quiet and controlled circumstances, but this was not always possible.

## Results

### Task Force Creation

Fourteen professionals were hired by Soleterre. Three worked remotely at the hotline, 11 worked in the ward. They totaled 1.912 h on the project (on average 32 per day).

### Population

One hundred and seven health care workers (44 males, mean age 40 ± 15) were recruited for the project. Their role, place of work and gender are summarized in Table 1. Sixteen missing or unspecified data are due either to mobilization of some staff across several ward, or to communication difficulties during extreme emergency conditions. Compared to the total number of health care workers involved in the first wave of the COVID-19 emergency, the study included 19.7% of ICU staff, 38.7% of Infectious Disease staff, 6.0% of Internal Medicine staff and 25.8% of Emergency Room staff. In total, 23.7% of the personnel involved in the emergency participated in the study. Participation was dictated by the self-perception of need combined with the help proposal of psychologists in a context of emergency debriefing and defusing. Those who denied participation did so mainly because they did not have time to spare or were not willing to share psychic pain (Table 2).

**TABLE 1:** Number of health care staff involved in the intervention according to role and place of work—number of males are in parentheses.

				Place of work				
	
			ICU	Infectious disease	Internal medicine	Emergency room	Non-specified	Total
Role	Physicians	Senior	14 (7)	11 (4)		2 (1)	2	39 (18)
		Junior	3 (2)	5 (2)	1 (1)	1 (1)		
		Non-specified						
	Nurses	Regular	21 (10)	3	2	9 (4)		40 (15)
		Head nurses	2					
		Bed Managers					1 (1)	
		Non-specified					2	
	Auxiliary nurses		1	4 (1)	2 (1)	2		9 (2)
	Unknown		3 (2)	1	1	3 (1)	11 (6)	19 (9)
	Total		44 (21)	24 (7)	6 (2)	17 (7)	16 (7)	107 (44)

**TABLE 2:** Number of health care staff involved in the study compared to the total number of staff normally employed.

	Total staff employed for COVID-19 emergency	Staff included in the study	% Staff included in the study compared to total staff employed for COVID-19 emergency
ICU	223	44	19.7
Infectious disease	62	24	38.7
Internal medicine	100	6	6.0
Emergency room	66	17	25.8
NN*		16	
**Total**	**451**	**107**	**23.7**

*Participants not assigned to a specific department because they worked in more than one department.

### Mental Health Assessment

One hundred and seven questionnaires were administered to 107 health care staff. In addition to the official surveys, it is possible to estimate about 280 monthly activations of psychologists for the benefit of health operators. This number, given the peculiarity of the context, includes both formal meetings and contacts in informal situations on the premises of the ward (during breaks or shift changes).

**FIGURE 1:**
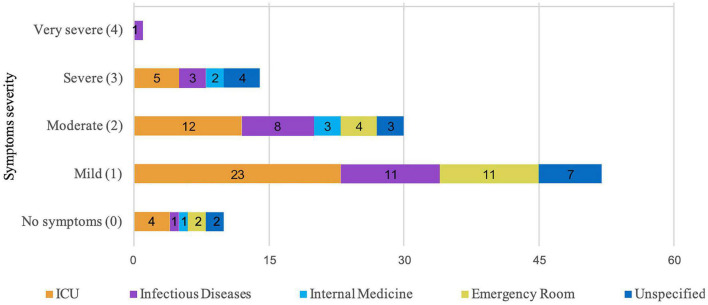
National stressful events survey PTSD short scale (NSESSS) results according to severity of symptoms and place of work.

Results of NSESSS according to severity of symptoms and place of work are represented in Figure 1. PTSD symptoms were reported as severe for 13% of the population. Most staff presented with mild (49%) and moderate (28%) symptoms of PTSD.

Results of DSM-5 Self-Rated Level 1 transversal Symptom Measure—Adult are represented in Table 3. Depressive symptoms were reported as severe for 7% of the population. Most staff presented with mild (35%), very mild (19%), and moderate (17%) symptoms of Depression.

**TABLE 3:** Severity of transversal symptoms in percentages.

Depression	Absent	22%
	Very mild	19%
	Mild	35%
	Moderate	17%
	Severe	7%
Anger	Absent	26%
	Very mild	12%
	Mild	30%
	Moderate	21%
	Severe	11%
Mania	Absent	19%
	Very mild	15%
	Mild	19%
	Moderate	25%
	Severe	22%
Anxiety	Absent	15%
	Very mild	21%
	Mild	21%
	Moderate	29%
	Severe	14%
Somatic symptoms	Absent	43%
	Very mild	19%
	Mild	13%
	Moderate	18%
	Severe	7%
Suicidal ideation	Absent	93%
	Very mild	4%
	Mild	0%
	Moderate	2%
	Severe	1%
Psychosis	Absent	90%
	Very mild	4%
	Mild	4%
	Moderate	1%
	Severe	2%
Sleep problems	Absent	32%
	Very mild	12%
	Mild	17%
	Moderate	20%
	Severe	20%
Memory	Absent	71%
	Very mild	13%
	Mild	11%
	Moderate	4%
	Severe	1%
Repetitive thoughts and behaviors	Absent	51%
	Very mild	20%
	Mild	15%
	Moderate	5%
	Severe	9%
Dissociation	Absent	76%
	Very mild	9%
	Mild	8%
	Moderate	6%
	Severe	1%
Personality functioning	Absent	49%
	Very mild	19%
	Mild	18%
	Moderate	10%
	Severe	4%

*Frequency of severity of transversal symptoms as assessed by the DSM-5 Self-Rated Level 1 transversal Symptom Measure—Adult. In accordance with the recommendations of DSM-5 Substance-Related Disorders Work Group, we did not consider the data relating to substances abuse due to the impossibility of providing a subdivision between the different types of substances.*

Anger symptoms were reported as severe for 11% of the population. Most staff presented with mild (30%), moderate (21%), and very mild (12%) symptoms of Anger.

Mania symptoms were reported as severe for 22% of the population. Most staff presented with moderate (25%), mild (19%), and very mild (15%) symptoms of Mania.

Anxiety symptoms were reported as severe for 14% of the population. Most staff presented with moderate (29%), mild (21%), and very mild (21%) symptoms of Anxiety.

Somatic symptoms were reported as severe for 7% of the population and absent for 43%, severe Suicidal Thoughts were experienced by 1% of the population and absent for 93%. Severe Psychotic symptoms were experienced by 2% of the population and absent for 90%.

Sleep Disorders were reported as severe for 20% of the population, most staff presented with moderate (20%), mild (17%), and very mild (12%) Sleep Disorders.

Memory Disorders were reported as absent for 71% and very mild for 13%.

Repetitive Thoughts and Behavior were reported as absent for 51% and very mild for 20%.

Dissociative Disorders were reported as absent for 76% and Personality Disorders were reported as absent for 49%, very mild for 19%, and mild for 18% of the population.

### Therapeutic Procedure

After the test administration, the results were assessed and those who had moderate and severe symptoms of PTSD were offered support (a maximum number of five individual session carried out by psychologists and psychotherapists who adopted a psychoanalytic orientation). Two hundred and seven individual sessions were offered (91 in the ICU, 55 in Infectious Diseases, 24 in Internal Medicine, 34 in the Emergency Room, and 3 non-specified).

## Conclusion

During the emergence of the COVID-19 pandemic, psychological intervention brought to light in health care workers feelings of grief, loss, uncertainty about the present and future, constant alarm with emotional and physical tension, helplessness and vulnerability.

As demonstrated by the literature from previous pandemic situations (e.g., SARS in 2003), we expect long-term adverse effects on the mental health of medical staff engaged in front-line care of COVID-19 patients (Lu et al., 2020; Xu et al., 2020).

While it is impossible to predict who will suffer from PTSD in a given situation, it is known that certain factors occurring before, in conjunction with, or after the event may contribute to the onset of this disorder (e.g., chronic exposure to traumatic events). Similarly, psychological intervention often turns out to be a protective factor for the onset of the disorder.

Acute stress reactions can be normal for frontline staff and with protective actions (e.g., psychological support) they usually resolve relatively quickly in the following months (Cole et al., 2020; Walton et al., 2020) when workers feel supported and understood by the organization that offers psychological support and coping and resilience strategies. In the literature, the interviews with American health workers held during the first week of the COVID-19 pandemic showed that the main concerns of the staff were to feel understood and supported on a psychological level (“a mental health professional takes care of me”) (Shanafelt et al., 2020). The British Psychological Society (2020) has highlighted formal counseling as a key way to support front-line staff. The data show that early psychological intervention is an important protective factor especially for grieving and PTSD (ITV News, 2020).

The possibility of acting upon the results of the TMHA COVID-19 CWs allowed an early intervention through individual session at least for moderate and severe PTSD as assessed by the NSESSS and symptom detection with the DSM-5 Self-Rated Level 1 transversal Symptom Measure—Adult. Therefore, although the data was collected at the time of maximum stress exposure we believe that our intervention represented a protective and support factor for mental well-being. At the end of the project, we registered increasing requests for the presence of the psychologists from the ward involved: the role of psychological support as a protective factor during the emergency period had been recognized and applied also during times of regular activity of the units.

The main difficulties we faced were the lack of a stable setting due to the emergency and the continuous “need of meaning” for the therapists. Seven hours of intervision were needed, conducted by a SPI (Italian Society of Psychoanalysis) and IPA (International Psychoanalytic Association) psychoanalyst trainer.

Since the project took place during the first phase of the emergency, there was also a high risk for the psychologists involved to contract COVID-19, however, thanks to prevention measures and the avail ability of PPE none of the equipe got infected.

The data collected during the first wave are in line with an Italian study conducted by Di Tella et al. (2020) with 145 health care workers (72 physicians and 73 nurses) that found higher levels of depressive and post-traumatic symptoms in physicians and nurses employed in front-line ward treating COVID-19 cases than in physicians and nurses working in non-COVID-19 ward.

The variety of roles and ward involved in this study is both a strength and a limitation, as the results represent all the staff majorly involved in the emergency but are consequently of little homogeneity. However, the study was activated during a time when the priority was catering to all the staff in a timely manner, and reaching all first-line worker was considered more ethical than differentiating the intervention on the basis of roles or workplace. Moreover, it worked as a starting point for the RCT on telematic/in person psychological sessions previously described.

Given our findings, we recommend implementing a mental health screening program in ward involved in COVID-19 patients’ care.

## Data Availability Statement

The raw data supporting the conclusions of this article will be made available by the authors, without undue reservation.

## Ethics Statement

The studies involving human participants were reviewed and approved by the Comitato Etico di Pavia. The patients/participants provided their written informed consent to participate in this study.

## Author Contributions

EA designed the project and wrote the manuscript. DR managed and supervised the project. AR was the project coordinator. FB was the research assistant. EP collected and analyzed the data and wrote the manuscript. AC, FM, and SP collected the data. RB supervised the project. All authors contributed to the article and approved the submitted version.

## Conflict of Interest

The authors declare that the research was conducted in the absence of any commercial or financial relationships that could be construed as a potential conflict of interest.

## Publisher’s Note

All claims expressed in this article are solely those of the authors and do not necessarily represent those of their affiliated organizations, or those of the publisher, the editors and the reviewers. Any product that may be evaluated in this article, or claim that may be made by its manufacturer, is not guaranteed or endorsed by the publisher.

## Footnotes

1 National Cancer Institute, Kyiv, Pediatric Department of the Romodanov Neurosurgery Institute, Kyiv, Western Ukrainian Specialized Children’s Medical Centre, Lviv, Hospital di Treichville, Abidjan, SS. Annunziata Hospital, Taranto, *IRCCS Policlinico San Matteo Foundation*, Pavia, Ibn Sina Centre of Paediatric Hematology and Oncology, Rabat, Centre of Paediatric Hematology and Oncology, Marrakesh, St Mary’s Lacor Hospital, Gulu Regional Referral Hospital (GRRH).

2 Morocco, Ukraine, Philippines, India, Ivory Coast Italy, El Salvador, Panama, Costa Rica, Nicaragua, Honduras, Guatemala, Belize, Mexico, Dominican Republic, Uganda, Senegal, Central African Republic, Sierra Leone, Democratic Republic of Congo, Syria, Burkina Faso.

## References

[B1] Andersson G. Cuijpers P. Carlbring P. Riper H. Hedman E. (2014). Guided Internet-based vs. face-to- face cognitive behavior therapy for psychiatric and somatic disorders: a systematic review and meta- analysis. *World Psychiatry* 13 288–295. 10.1002/wps.20151 25273302 PMC4219070

[B2] Asperges E. Novati S. Muzzi A. Biscarini S. Sciarra M. Lupi M. (2020). COVID-19 IRCCS policlinico san matteo foundation task force. Rapid response to COVID-19 outbreak in Northern Italy: how to convert a classic infectious disease ward into a COVID-19 response centre. *J. Hosp. Infect.* 105 477–479. 10.1016/j.jhin.2020.03.020 32205162 PMC7118420

[B3] Berryhill M. B. Culmer N. Williams N. Halli-Tierney A. Betancourt A. Roberts H. (2019a). Vid- eoconferencing psychotherapy and depression: a systematic review. *Telemed. J. E Health* 25 435–446. 10.1089/tmj.2018.0058 30048211

[B4] Berryhill M. B. Halli-Tierney A. Culmer N. Williams N. Betancourt A. King M. (2019b). Vid- eoconferencing psychological therapy and anxiety: a systematic review. *Family Pract.* 36 53–63. 10.1093/fampra/cmy072 30188992

[B5] Carmassi C. Foghi C. Dell’Oste V. Cordone A. Bertelloni C. A. Bui E. (2020). PTSD symp- toms in healthcare workers facing the three coronavirus outbreaks: what can we expect after the COVID-19 pandemic. *Psychiatry Res.* 292:113312. 10.1016/j.psychres.2020.113312 PMC7370915 32717711

[B6] Cole C. L. Waterman S. Stott J. Saunders R. Buckman J. E. J. Pilling S. (2020). Adapting IAPT services to support frontline NHS staff during the Covid-19 pandemic: the Homerton covid psycho- logical support (HCPS) pathway. *Cogn. Behav. Ther.* 13:e12. 10.1017/S1754470X20000148 32454891 PMC7235312

[B7] Di Tella M. Romeo A. Benfante A. Castelli L. (2020). Mental health of healthcare workers during the COVID-19 pandemic in Italy. *J. Eval. Clin. Pract.* 26 1583–1587. 10.1111/jep.13444 32710481

[B8] Gentry M. T. Lapid M. I. Clark M. M. Rummans T. A. (2019). Evidence for telehealth group-based treatment: a systematic review. *J. Telemed. Telecare* 25 327–342. 10.1177/1357633X18775855 29788807

[B9] Greenberg N. Weston D. Hall C. Caulfield T. Williamson V. Fong K. (2021). Mental health of staff working in intensive care during COVID-19. *Occup. Med.* 71 62–67. 10.1093/occmed/kqaa220 33434920 PMC7928568

[B10] ITV News (2020). *Mental Health ‘Damaged’ in Majority of Scottish Coronavirus Carers.* London: ITV News.

[B11] Lenti M. V. Corazza G. R. Di Sabatino A. (2020). Carving out a place for internal medicine during COVID-19 epidemic in Italy. *J. Internal Med.* 288 263–265. 10.1111/joim.13079 32294269 PMC7262077

[B12] Liu S. Yang L. Zhang C. Xiang Y. T. Liu Z. Hu S. (2020). Online mental health services in China during the COVID-19 outbreak. *Lancet Psychiatry* 7 e17–e18. 32085841 10.1016/S2215-0366(20)30077-8 PMC7129099

[B13] Lu W. Wang H. Lin Y. Li L. (2020). Psychological status of medical workforce during the COVID-19 pandemic: a cross-sectional study. *Psychiatr. Res.* 288:112936. 10.1016/j.psychres.2020.112936 PMC7195354 32276196

[B14] Maunder R. Hunter J. Vincent L. Bennett J. Peladeau N. Leszcz M. (2003). The immediate psychological and occupational impact of the 2003 SARS outbreak in a teaching hospital. *Can. Med. Assoc. J.* 168 1245–1251. 12743065 PMC154178

[B15] Perlini S. Canevari F. Cortesi S. Sgromo V. Brancaglione A. Contri E. (2020). COVID19 IRCCS policlinico san matteo foundation task force. Emergency Department and Out-of-Hospital Emergency Sys- tem (112-AREU 118) integrated response to Coronavirus Disease 2019 in a Northern Italy centre. *Internal Emerg. Med.* 15 825–833. 10.1007/s11739-020-02390-4 32507926 PMC7276336

[B16] Santesteban-Echarri O. Piskulic D. Nyman R. K. Addington J. (2020). Telehealth interventions for schiz- ophrenia-spectrum disorders and clinical high-risk for psychosis individuals: a scoping review. *J. Telemed. Telecare* 26 14–20. 10.1177/1357633X18794100 30134781

[B17] Shanafelt T. Ripp J. Trockel M. (2020). Understanding and addressing sources of anxiety among health care professionals during the COVID-19 pandemic. *J. Am. Med. Assoc.* 323 2133–2134. 10.1001/jama.2020.5893 32259193

[B18] Shore J. H. Hilty D. M. Yellowlees P. (2007). Emergency management guidelines for telepsychiatry. *Gen. Hosp. Psychiatry* 29 199–206. 10.1016/j.genhosppsych.2007.01.013 17484936 PMC1986661

[B19] Siddiqi H. K. Mehra M. R. (2020). COVID-19 illness in native and immunosuppressed states: a clinical- therapeutic staging proposal. *J. Heart Lung Transplant.* 39 405–407. 10.1016/j.healun.2020.03.012 32362390 PMC7118652

[B20] The British Psychological Society (2020). *The Psychological Needs of Healthcare Staff as a result of the Coronavirus Pandemic.* Leicester: The British Psychological Society.

[B21] Walton M. Murray E. Christian M. D. (2020). Mental health care for medical staff and affiliated healthcare workers during the COVID-19 pandemic. *Eur. Heart J. Acute Cardiovasc. Care* 9 241–247. 10.1177/2048872620922795 32342698 PMC7189614

[B22] West C. P. Dyrbye L. N. Shanafelt T. D. (2018). Physician burnout: contributors, consequences and solutions. *J. Intern. Med.* 283 516–529. 10.1111/joim.12752 29505159

[B23] Xu J. Xu Q. Wang C. Wang J. (2020). Psychological status of surgical staff during the COVID- 19 outbreak. *Psychiatr. Res.* 288:112955. 10.1016/j.psychres.2020.112955 32302815 PMC7151272

