# Supplementary material for: Psychological Support in a COVID-19 Hospital: A Community Case Study
Source: Front Psychol. 2022 Feb 17;12:820074. doi: 10.3389/fpsyg.2021.820074 (PMC8893142; doi:10.3389/fpsyg.2021.820074)
Supplement: Supplementary file 1 [file Data_Sheet_1.PDF]

Therapeutic Model for COVID-19

MENTAL SYMPTOMS

The construct was created by a group of clinical psychologists from the Soleterre Foundation active at "IRCCS Policlinico San Matteo" of Pavia to support patients, relatives, doctors, and nurses by borrowing the medical strategies of COVID-19 disease's approach, at clinical summits, starting from the following assumptions:

- It is a new disease that needs a new approach (we cannot treat it as "similar to a disease we already know"), **thus preparing new clinical criteria**;
- Starting from the **rheumatology model** which considers 2 phases a) viral b) immune response, in the same way at the level of mental symptomatology it is expected a) a potential trauma ("viral" element) b) a potential readjustment ("immune response" element). If no re-adaptation occurs, the onset of pathological symptoms linked to the specific life experience of the individual is very likely.
- Thinking about hospital and territorial management (also for non-hospitalized people) of the effects of the COVID-19 threat on the population.

The COVID-19 threat scenario concerns the entire population, hospitalized relatives and patients and healthcare personnel. Everyone, with different intensity, is involved. We are therefore talking about a collective, social threat. Unlike the perception of the individual threat impression, the **perception of collective (social) threat** assumes the characteristic of:

1) **rapid transformation of the rules** that until a moment before ruled group interdependence concerning the essential characteristics of life and death.
2) **request to take rapid decisions** (to scientists, decision-makers, communicators, and citizens)
3) **feeling to have fewer resources than needed** to face the emergency.
4) activation of a repertoire of **emotions appropriate** to the context (fear, anger, helplessness...).

for field workers and hospitalized patients and family members are added

5) **mourning, loss, traumatic events** with loss of certainty about the present and the future, constant alert with emotional and physical tension. Recurring feelings of helplessness and vulnerability.

While it is understandable that healthcare workers and the hospitalized population are more exposed to traumatic situations, it is also likely that the social life restrictions' effects (home quarantine and interruption of normal activities at work, at school...) have a high potential in terms of loss of our certainties in the present and the future, and being constantly alarmed by emotional and physical tension. Recurring feelings of helplessness and vulnerability.

The intensity and quality of the negative outcomes of a potentially traumatic situation are the result of the balance between event's characteristics and protection's factors.

What are these protective factors? Here are some examples:

1) Talking about your state, how you feel. A non-recorded experience remains unprocessed.
2) To activate behaviors of affiliation and intimacy, of collaboration and mutual help. (Neurosciences have highlighted the relevance of intercerebral interactions, of affective transactions with other brains and therefore the importance of relational security that the subject experiences)
3) Consult a mental health professional

Protective factors are those that put our minds in a safe place, " in safety". The concept of security must be placed at the center of our thoughts concerning potentially traumatic experiences and fear that constantly stress our defense systems against threats. The need for security is for all of us a basis for mental and physical health.

Today we are dealing with an entirely frightened society that feels insecure and risks using all its energies in defense. If security is lacking our energy is committed to defense.

The "COVID-19 therapeutic algorithm" model starts from the assumption that the whole population, during the Covid.-19 emergency period is potentially affected by disorders related to life events and stressful conditions, including dissociative disorders (also following the historical relation between dissociation and conversion) maintained in the ICD-10 (see algorithm and table).

In detail, during the emergency phase, we consider these defenses the "typical" ones in front of a threat situation, that can be grouped in 3 large areas:

Hyperactivation, a permanent state of alert that aims to “unload”, “live at full speed”, “don’t lose anything” and thus have the feeling of “keeping things under control” while the whole world around us loses elements of daily certainty. Typical thoughts can be “I’m in danger”, “I’m bursting”, “this is a unique opportunity, but… if I lose, I lose everything…”.
In such hyperactive behaviour (symptoms of hyperarousal) the psychophysical system increases the level of excitement and amplifies the emotional instability, and exaggerated fear or aggressiveness.

Turning off, a state of demotivation, withdrawal. A sort of loss of energy that leads us to live at a minimum, in the “burrow” with thoughts like “I am worth nothing”, “I am a burden”, “nothing is worth it”, “I have no future”, “we’ll see and when the situation returns less dangerous we’ll think about what to do”.
In such a hypoactivity behaviour (symptoms of hypoarousal) the psychophysical system lowers the level of excitement and attenuates the alarm reaction, which could cause hypersomnia and insensitivity to stress.

> **Dissociation,** a kind of interruption between us and the threatening event. Not thinking about it and with the conviction that “nothing is true”, “Eye can’t see, heart doesn’t hurt!”, “I’ll pull the plug, what’s the problem anyway?”, “If I don’t think about it, I feel better.”
> In this dissociative behaviour (dissociative symptoms) the psychophysical system acts a sort of interruption of the generally integrated functions of consciousness, memory, identity or awareness of the body, the self or the environment.

All three behaviors are defensive ones and are fine in a moment of alarm.
However, it is necessary to be careful that they do not become chronic behaviors (to get chronic in a noradrenergic dysfunction), going ahead for a period that goes beyond the threat (in the post-coronavirus emergency to be precise) because they could turn from defenses into pathogenic behaviors.

Freud wrote in 1914 that the effect of trauma on the individual is to **alter a) the foundations and b) the contours of his life and to compromise the person's ability to live in the present, which is due to continuous efforts to reconcile with the trauma experienced and the impact that experience has on the present and the future**. Traumatic events have the characteristic to exceed the individual's ability to adapt to them and cause a state of despair.

According to contemporary researchers, the fact of being overwhelmed by an event has a biological correlation in brain structures whose functions are altered, and together with these, there are different physiological responses in individuals who face the trauma and develop PTSD. In this sense, peritraumatic dissociation (at the time of the trauma or immediately afterward) has been identified as a risk factor for the subsequent development of PTSD. If dissociation can be protective at the time of trauma, it compromises the individual's ability to process the trauma later and therefore leads to the development of PTSD.

Therefore, we believe that as for the whole community there will be a moment of "return to life", once overcame the peak and reached the plateau that brings us (symbolically / and practically) outside the emergency, at the individual level each person will have a specific life history and a sort of "individual peak" for the return to his or her possible life. This may not be all dissimilar to the one before the emergency or on the contrary very different.

It will, therefore, be necessary to foresee from a potentially traumatic phase to a phase of adaptation that for each individual or group will have different moments in time and the way of transition.

Therapeutic Model for COVID-19
MENTAL SYMPTOMS

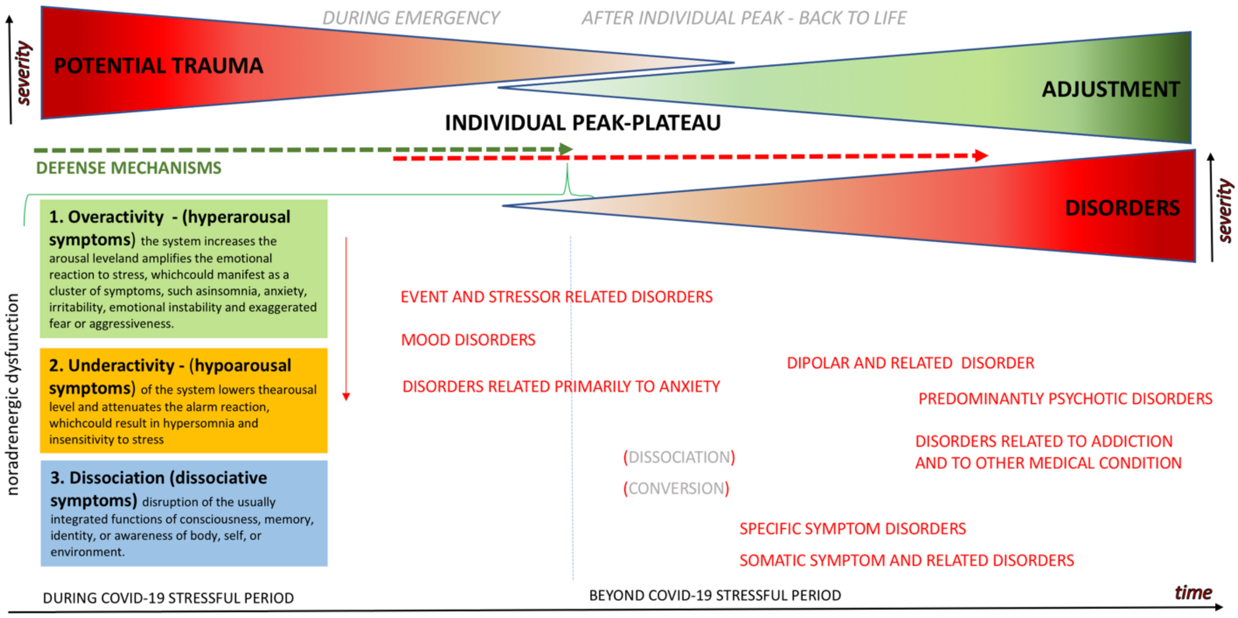

Therapeutic Model for COVID-19
MENTAL SYMPTOMS

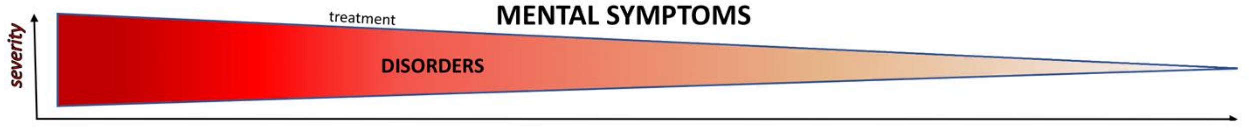

DSM-5

Trauma- and stressor-related disorders
- F43.10. Posttraumatic stress disorder
- F43.0. Acute stress disorder
- F43.2. Adjustment disorders
  - .21. With depressed mood
  - .22. With anxiety
  - .23. With mixed anxiety and depressed mood
  - .24. With disturbance of conduct
  - .25. With mixed disturbance of emotions and conduct

Dissociative disorders
- F44.81. Dissociative identity disorder
- F44.0. Dissociative amnesia. *Specify if:*
- F44.1. With dissociative fugue
- F48.1. Depersonalization/derealization disorder
- F44.88. Other specified dissociative disorder (OSDD)
- F44.81. OSDD-1, chronic and recurrent syndromes of mixed dissociative symptoms
- F44.88. OSDD-2, identity disturbance due to prolonged and intense coercive persuasion
- F44.88. OSDD-3, acute dissociative reactions to stressful events
- F44.3. OSDD-4, dissociative trance
- F44.9. Unspecified dissociative disorder\

Somatic symptom and related disorders
- F44. Conversion disorder
  - .4. With weakness or paralysis
  - .4. With abnormal movement
  - .4. With swallowing symptoms
  - .4. With speech symptoms
  - .5. With attacks or seizures
  - .6. With anesthesia or sensory loss
  - .6. With special sensory symptom
  - .7. With mixed symptoms

Somatic symptom and related disorders
- F45.1. Somatic symptom disorder
- F45.21. Illness anxiety disorder
- F68.10. Factitious disorders
- F45.8/.9. Other specified/unspecified somatic symptom and related disorder

ICD-10

F40-48. Neurotic, stress-related, and somatoform disorders
- F43. Reaction to severe stress, and adjustment disorders
  - .0. Acute stress reaction
  - .1. Posttraumatic stress disorder
  - .2. Adjustment disorders
  - .20. Brief depressive reaction
  - .21. Prolonged depressive reaction
  - .22. Mixed anxiety and depressive reaction
  - .23. With predominant disturbance of other emotions
  - .24. With mixed disturbance of emotions and conduct

Dissociative (conversion) disorders
- F44.0. Dissociative amnesia
- F44.1. Dissociative fugue
- F44.3. Trance and possession disorders
- F44.7. Mixed dissociative (conversion) disorders
- F44.8. Other dissociative (conversion) disorders
  - .80. Ganser's syndrome
  - .81. Multiple personality disorder
  - .88. Other specified dissociative (conversion) disorders
- F44.9. Dissociative (conversion) disorder, unspecified
- F48. Other neurotic disorders
- F48.1. Depersonalization-derealization syndrome
- F44. Dissociative (conversion) disorders
  - .2. Dissociative stupor
  - .4. Dissociative motor disorders
  - .5. Dissociative convulsions

F45. Somatoform disorders
- .0. Somatization disorder
- .1. Undifferentiated somatoform disorder
- .2. Hypochondriacal disorder
- .3. Somatoform autonomic dysfunction
- .4. Persistent somatoform pain disorder
- .8/.9. Other specified/Unspecified somatoform disorders

F68. Other disorders of adult personality and behavior
- .1. Intentional production or feigning of symptoms or disabilities, either physical or psychological (factitious disorder)

PDM-2

S4 Event- and stressor-related disorders
- S41 Trauma- and stressor-related disorders
  - S41.1 Adjustment disorders
  - S41.2 Acute and posttraumatic stress disorders
  - S41.3 Complex posttraumatic stress disorder
- S42 Dissociative disorders
  - S42.1 Depersonalization/derealization disorder
  - S42.2 Dissociative amnesia ± fugue
  - S42.3 Dissociative identity disorder and other specified dissociative disorder-1
- S43 Conversion disorder

S5 Somatic symptom and related disorders
- S51 Somatic symptom disorder
- S52 Illness anxiety disorder (Hypochondriasis)
- S53 Factitious disorders

DURING AND BEYOND COVID-19 STRESSFUL PERIOD

Clinical cases collection

The following data should be collected (during interview) for each patient seen in the emergency period (before collective peak and recovery):

Severity of post-traumatic stress symptoms

National Stressful Events Survey PTSD Short Scale (NSESSS)

Name:_______________________ **Age:**______ **Sex:** ☐**Male** ☐**Female** **Date:**____________

Please list the traumatic event that you experienced:____
Date of the traumatic event:_________________________

Instructions: People sometimes have problems after extremely stressful events or experiences. How much have you been bothered during the PAST SEVEN (7) DAYS by each of the following problems that occurred or became worse after an extremely stressful event/experience? **Please respond to each item by marking (v or x) one box per row.**

							Clinician Use
		Not at all	A Little bit	Moderately	Quite a bit	Extremely	Item score
1.	Having "flashbacks," that is, you suddenly acted or felt as if a stressful experience from the past was happening all over again (for example, you reexperienced parts of a stressful experience by seeing, hearing, smelling, or physically feeling parts of the experience)?	❑ 0	❑ 1	❑ 2	❑ 3	❑ 4	
2.	Feeling very emotionally upset when something reminded you of a stressful experience?	❑ 0	❑ 1	❑ 2	❑ 3	❑ 4	
3.	Trying to avoid thoughts, feelings, or physical sensations that reminded you of a stressful experience?	❑ 0	❑ 1	❑ 2	❑ 3	❑ 4	
4.	Thinking that a stressful event happened because you or someone else (who didn't directly harm you) did something wrong or didn't do everything possible to prevent it, or because of something about you?	❑ 0	❑ 1	❑ 2	❑ 3	❑ 4	
5.	Having a very negative emotional state (for example, you were experiencing lots of fear, anger, guilt, shame, or horror) after a stressful experience?	❑ 0	❑ 1	❑ 2	❑ 3	❑ 4	
6.	Losing interest in activities you used to enjoy before having a stressful experience?	❑ 0	❑ 1	❑ 2	❑ 3	❑ 4	
7.	Being "super alert," on guard, or constantly on the lookout for danger?	❑ 0	❑ 1	❑ 2	❑ 3	❑ 4	
8.	Feeling jumpy or easily startled when you hear an unexpected noise?	❑ 0	❑ 1	❑ 2	❑ 3	❑ 4	
9.	Being extremely irritable or angry to the point where you yelled at other people, got into fights, or destroyed things?	❑ 0	❑ 1	❑ 2	❑ 3	❑ 4	
						**Total/Partial Raw Score:**	
						**Prorated Total Raw Score: (if 1-2 items left unanswered)**	
						**Average Total Score:**	

Kilpatrick DG, Resnick HS, Friedman, MJ. Copyright © 2013 American Psychiatric Association. All rights reserved.
This measure can be reproduced without permission by researchers and by clinicians for use with their patients.

Level 1 cross-cutting symptom evaluation scale

Name:________________________ **Age:**______ **Sex:** ☐**Male** ☐**Female** **Date:**_____________

If this questionnaire is completed by an informant, **what is your relationship with the individual?**__________
In a typical week, approximately how much time do you spend with the individual? _________hours/week

Instructions: The questions below ask about things that might have bothered you. For each question, circle the number that best describes how much (or how often) you have been bothered by each problem during the **past TWO (2) WEEKS**.

	During the past **TWO (2) WEEKS**, how much (or how often) have you been bothered by the following problems?	**None** Not at all	**Slight** Rare, less than a day or two	**Mild** Several days	**Moderate** More than half the days	**Severe** Nearly Every day	**Highest Domain Score** (clinician)
I.	1. Little interest or pleasure in doing things?	0	1	2	3	4	
	2. Feeling down, depressed, or hopeless?	0	1	2	3	4	
II.	3. Feeling more irritated, grouchy, or angry than usual?	0	1	2	3	4	
III.	4. Sleeping less than usual, but still have a lot of energy?	0	1	2	3	4	
	5. Starting lots more projects than usual or doing more risky things than usual?	0	1	2	3	4	
IV.	6. Feeling nervous, anxious, frightened, worried, or on edge?	0	1	2	3	4	
	7. Feeling panic or being frightened?	0	1	2	3	4	
	8. Avoiding situations that make you anxious?	0	1	2	3	4	
V.	9. Unexplained aches and pains (e.g., head, back, joints, abdomen, legs)?	0	1	2	3	4	
	10. Feeling that your illnesses are not being taken seriously enough?	0	1	2	3	4	
VI.	11. Thoughts of actually hurting yourself?	0	1	2	3	4	
VII.	12. Hearing things other people couldn't hear, such as voices even when no one was around?	0	1	2	3	4	
	13. Feeling that someone could hear your thoughts, or that you could hear what another person was thinking?	0	1	2	3	4	
VIII.	14. Problems with sleep that affected your sleep quality over all?	0	1	2	3	4	
IX.	15. Problems with memory (e.g., learning new information) or with location (e.g., finding your way home)?	0	1	2	3	4	
X.	16. Unpleasant thoughts, urges, or images that repeatedly enter your mind?	0	1	2	3	4	
	17. Feeling driven to perform certain behaviors or mental acts over and over again?	0	1	2	3	4	
XI.	18. Feeling detached or distant from yourself, your body, your physical surroundings, or your memories?	0	1	2	3	4	
XII.	19. Not knowing who you really are or what you want out of life?	0	1	2	3	4	
	20. Not feeling close to other people or enjoying your relationships with them?	0	1	2	3	4	
XIII.	21. Drinking at least 4 drinks of any kind of alcohol in a single day?	0	1	2	3	4	
	22. Smoking any cigarettes, a cigar, or pipe, or using snuff or chewing tobacco?	0	1	2	3	4	
	23. Using any of the following medicines ON YOUR OWN, that is, without a doctor's prescription, in greater amounts or longer than prescribed [e.g., painkillers (like Vicodin), stimulants (like Ritalin or Adderall), sedatives or tranquilizers (like sleeping pills or Valium), or drugs like marijuana, cocaine or crack, club drugs (like ecstasy), hallucinogens (like LSD), heroin, inhalants or solvents (like glue), or methamphetamine (like speed)]?	0	1	2	3	4	

Copyright © 2013 American Psychiatric Association. All Rights Reserved.
This material can be reproduced without permission by researchers and by clinicians for use with their patients.

Evaluating the impact of symptoms on life history

1) Me before the health emergency...

2) Then what happened was...

3) Now I look around and see...

4) I feel...

5) And I think...

6) My days...

7) The people around me...

8) The others...

9) At home...

10) The things that make me feel better...

11) From this experience I am learning...

12) For tomorrow I imagine...

13) And I would like...

Note on the realization

The Model was realized in March 2020 by a small group of psychologists (Dr. Rizzi Damiano, Dr. Anna Rovati and Dr. Ivan Giacomel) with the collaboration of an SPI psychiatrist psychoanalyst Dr. Fulvio Mazzacane. An enlarged working group composed of psychologists Dr. Massimo Buratti, Dr. Matteo Mangiagalli, Dr. Anna Cinquini, Dr. Monica Demichelis, Dr. Federico Scimone, Dr. Giulia Sollazzo, Dr. Martina Tosi, tested the model to the general population, to the patients' families and to doctors and nurses in the First Aid, Infectious Diseases and ICU wards of the IRCCS "Policlinico San Matteo" Hospital of Pavia.
The support of the Institute of Child and Adolescent Psychotherapy of Milan (PSIBA) was fundamental for the realization.
Special thanks to Prof. Francesco Mancuso, Prof. Piergiorgio Tagliani, Prof.ssa Elena Pizzi and Prof.ssa Paola Picozzi.

Thanks to dott.ssa Caterina Galandra for the collaboration in implementing the protocol for data collection about participants.

The work is under a phase of testing with an international survey thanks to an agreement with La Sapienza University in Rome, Dipartimento di Psicologia Dinamica e Clinica. Special thanks to Prof.ssa Velotti Patrizia.

Usage agreement

This model is available to the scientific community free of charge. Those who wish to use it are kindly requested to fill in and send the letter of request on the next page.

Dear Soleterre Foundation NGO/ONLUS,

Herewith the undersigned

____

____

____

intends to administer the questionnaires and monitor the individuals tested according to the **Therapeutic Model for COVID-19 MENTAL SYMPTOMS.**

Data will be collected anonymously but following must be specified:

- Sex
- Age
- Profession
- Interviewed place of residence
